# Diabetes in Shenzhen, China: epidemiological investigation and health care challenges

**DOI:** 10.7189/jogh.07.011102

**Published:** 2017-06

**Authors:** Xinfeng Yan, Hui Xia, Haitao Li, Xiaoting Deng, Lizhen Yang, Shaojuan Zhao, Jianfeng Zou, Yi Luo, Sijing Cao

**Affiliations:** 1 Longhua District Center for Chronic Disease Prevention and Control, Shenzhen, China; 2 School of Medicine, Shenzhen University, Shenzhen, China; * Joint first authorship

## Abstract

**Background:**

Understanding epidemiological characteristics of diabetes in a specific population will potentially benefit prevention and control of diabetes and policy–making. This study aimed to investigate the prevalence and awareness of diabetes, as well as its pharmacological, non–pharmacological and primary care management in Shenzhen, China.

**Methods:**

A cross–sectional study was conducted. We employed multistage cluster random sampling methods to select the participants. Face–to–face interview surveys were conducted to collect data. A total of 1676 participants completed the survey.

**Results:**

We found that the prevalence of diabetes was 4.8%. The prevalence of impaired fasting blood glucose was 6.0%. The prevalence rates of both diabetes and impaired fasting blood glucose increased with age (*P* < 0.001), whereas hypertension was strongly associated with diabetes only (odds ratio (OR) = 1.93, 95% confidence interval (CI) 1.15–3.22). The awareness of diabetes was poor (51.9%) and 54.3% of diabetic patients were not being treated pharmacologically. Less than one–third of diabetic patients were undergoing non–pharmacological treatments. Primary care management of diabetes was recorded for only 11.1% of the patients.

**Conclusions:**

Although diabetes prevalence in Shenzhen is about a half that of the Chinese average, high prevalence of impaired fasting blood glucose imposes a public health threat and burden to the health care system. Approximately half of the subjects with diabetes are undiagnosed. Our findings highlight the need of public health efforts for primary and secondary prevention, as well as early detection of diabetes. Primary care may be crucial an improved access to medical services and better management of diabetes.

Diabetes is associated with increased mortality from a range of cardiovascular and non–cardiovascular diseases [1]. Statistics from the World Health Organization (WHO) show a rapid increase of diabetes prevalence during the past several decades. The current estimate of diabetes prevalence is 9% worldwide [2]. In 2014, diabetes caused 1.5 million deaths, with low– and middle–income countries disproportionately affected [3,4]. Therefore, diabetes represents a major public health concern worldwide, especially for developing countries [5].

In China, diabetes has also emerged as an important public health problem. Over the past several decades, diabetes prevalence increased sharply, from 0.7% in 1980 [6], to 2.7% in 2002 [7], to 11.6% in 2010 [8]. This implies that China is home to the largest diabetic population in the world. Statistics in 2013 showed that approximately one–fourth of worldwide diabetes–related deaths occurred in China [3]. However, there have been no obvious improvements in diabetes awareness, treatment and control, which are crucial to decrease its related complications and its financial burden [9].

Studies have shown that prevalence, awareness, management of diabetes, as well its risk factors are dependent on economy, culture and living regions etc. [10–12]. Shenzhen, China’s first Special Economic Zone holding sub–provincial administrative status, situates in the Pearl River Delta region of southern China. Shenzhen is a migrant city with about 70% of its population being migrants living within a total area of 1996.8 km^2^. Shenzhen is an important economic powerhouse, and represents one of the most developed area in China. Understanding epidemiological characteristics of diabetes in a specific population will potentially benefit the prevention and control of diabetes and policy–making. The current study aimed to investigate the prevalence and awareness of diabetes, as well as its pharmacological, non–pharmacological and primary care management in Shenzhen, China.

## METHODS

### Ethics

This study was approved by the Shenzhen Longhua District Center for Chronic Diseases Prevention and Control Ethics Committee.

### Study population

This cross–sectional study was a community–based household population survey conducted between April and May 2015. The study included subjects living in Shenzhen ≥6months in the past one year before the survey was performed and aged 18–70 years. Those living in institutions like nursing homes, and members of the regular Chinese Forces, were excluded. Using the formula *n = deff *×* u^2^ *×* p(1 – p)/d^2^*, where *deff* = 1.5 and *p* = 0.05, we calculated the sample size of 1752 for a 95% confidence level and 2.5% confidence interval. The final sample size was 2000, taking into consideration a 10% non–response rate. This study sampled the participants using a multistage cluster random sampling design. Two of the ten districts were first randomly selected using a simple random sampling approach. Ten neighborhoods were then randomly drawn from each randomly selected district. A total of 20 clusters were randomly selected. All dwellings in each neighborhood were listed and households were sampled employing a systematic random sampling method. The total number of households selected from each district was proportional to the population size of each district. Households were evenly distributed in each neighborhood stratified by district. Each household was contacted to obtain the list of current household members. A Kish method was adopted for participant selection within each household. The overall response rate was 89%.

### Data collection procedure

Data were collected using the World Health Organization (WHO) STEPS approach to chronic disease risk factor surveillance [13], which included a questionnaire on socio–demographic characteristics, clinical measurements and a subsequent blood sample for assessment of biochemical parameters. Face–to–face interview survey was adopted for the collection of socio–demographic factors and clinical measurements. The survey was conducted by specially trained interviewers. The participants were assured of anonymity and confidentiality of the survey, and informed consent was obtained before the survey was commenced. The participants were asked about their age, education level, occupation, marital status, registration, monthly household income. The participants were also asked, “Do you smoke in the past month?”, “Do you have diabetes diagnosed by a health professional?”, “What kind of pharmacological or non–pharmacological approaches are taken for management of diabetes?”, and “Are you under primary care management?”

During the interview, anthropometric measurements were obtained. Body weight was measured to the nearest 0.1 kg using a digital scale, and height to the nearest 0.1 cm in the standing position with a portable stadiometer. According to the protocol recommended by the national guidelines for hypertension management, blood pressure was measured using calibrated mercury sphygmomanometer. Two measurements were performed. Systolic blood pressure (SBP) and diastolic blood pressure (DBP) were recorded as the means of two measurements. If the difference between the two measurements was larger than 5 mm Hg, an additional measurement was performed and the mean of all three measurements would be recorded. On an appointed date after the interview, blood sample was obtained from participants. Twelve–hour fasting blood glucose levels were assessed according to WHO standardized fingertip prick tests, using calibrated blood glucose meters and reagent trips.

### Key definitions

Diabetes was defined as fasting blood glucose (FBG) ≥7.0 mmol/L, and/or self–reported physician–diagnosed condition, and/or participants’ reported drug treatment for diabetes currently. Impaired fasting blood glucose was defined as 5.6 mmol/L≤FBG<7.0 mmol/L.

Awareness of diabetes referred to participants’ self–report of any previous diagnosed condition by health professionals, and/or the use of insulin or medication for diabetes.

Pharmacological management was defined as a participant’s report of medication use for diabetes regularly or insulin injection for diabetes.

Non–pharmacological management was defined as changing diet, and/or engaging in exercise, and/or monitoring blood glucose regularly.

Controlled diabetes was defined as FBG<7.0 mmol/L.

### Descriptive variables

Socio–demographic information included age, gender, marital status, registration, education level, occupation, monthly household income. The participants were classified into the migrants and the locals, according to the registration. Migrants were defined as individuals who moved to a new location without changing their official Hukou registration [14]. Monthly household income was categorized into three groups according to the monthly household poverty line (RMB 5000, US$ 725) and mean monthly household income level (RMB10 000, US$ 1450) in Shenzhen in 2011 [15]. Lifestyle factors included the body mass index, self–reported smoking status and hypertension. Overweight and obesity were defined as an individual’s body mass index (BMI) of 24.0–27.9 kg/m^2^ and ≥28.0 kg/m^2^, respectively, whereas the BMI of ≤18.4 kg/m^2^ and 18.5–23.9 kg/m^2^ indicated underweight and normal weight, respectively [16]. Current smoking was self–reported and included individuals who smoke occasionally or daily. Hypertension was defined as self–reported physician–diagnosed condition and currently under antihypertensive treatment, and/or systolic blood pressure (SBP) ≥140mmHg and/or diastolic blood pressure (DBP) ≥90 mm Hg.

### Statistical analysis

Socio–demographic characteristics and lifestyle factors of participants were presented as percentages or means (SD). Prevalence estimates of impaired fasting blood glucose and diabetes were computed according to socio–demographic and lifestyle characteristics. χ^2^–tests were performed for comparison between participants with different socio–demographic and lifestyle characteristics. Two multivariate logistic regression models were constructed for the calculation of odds ratios (ORs) and 95% confidence interval (CI) to estimate the strength of associations between socio–demographic and lifestyle factors and impaired fasting blood glucose and diabetes. Model fittings were conducted using backward elimination, with a threshold of 0.10 for variable inclusion in the model. Awareness, management and control of diabetes were presented as prevalence rates. A *P* value <0.05 was considered statistically significant. All analyses were performed by using the SPSS 19.0 software (SPSS Inc., Chicago, IL, USA).

## RESULTS

### Characteristics of participants

Approximately three–fourths of the participants were aged between 18 to 44 years, and migrants. More than half of participants were women. The majority of participants were married or living with a partner (88.1%). Around one–third of the participants had middle-school education, and just over 10% had primary school or below. More than one–third of the participants were in the middle–income group, whereas 33.1% of participants rejected to answer the question or did not know their monthly household income. Mean SBP was 119.81 mm Hg, while mean DBP was 77.63 mm Hg. The prevalence of hypertension was 17.6%. Mean BMI was 23.50 kg/m^2^. Around two–fifths of the participants were overweight or obese. Approximately one–fifth of the participants were current smokers. Mean FBG was 4.81 mmol/L (**Table 1**).

**Table 1:** Characteristics of participants

Characteristics	No.	Unweighted %
**Age** (years), mean (SD):	1675	39.26 (11.13)
18–44	1179	70.3
45–59	393	23.4
≥60	103	6.1
**Gender:**		
Male	791	47.2
Female	885	52.8
**Registration:**		
Locals	442	26.4
Migrants	1216	72.6
**Marital status:**		
Never in union	149	8.9
Married or living with partner	1476	88.1
Widowed, divorced and separated	42	2.5
**Education:**		
Primary school and below	231	13.8
Middle school	603	36.0
High school and equivalent	528	31.5
3–year college and above	306	18.3
**Occupation:**		
Manual workers	253	15.1
Sales and services	320	19.1
Professional, technical and managerial	214	12.8
Clerical	176	10.5
Other workers	251	15.0
Not working	456	27.2
**Household income:**		
Low	170	10.1
Middle	606	36.2
High	346	20.6
Rejected	172	10.3
Do not know	382	22.8
**Hypertension:**		
Yes	295	17.6
No	1381	82.4
SBP, mean (SD)	1676	119.81 (15.65)
DBP, mean (SD)	1676	77.63 (10.79)
**BMI, mean (SD)**	1665	23.50 (3.71)
Underweight/normal weight	996	59.4
Overweight/obese	680	40.6
**Current smoking:**		
Yes	366	21.8
No	1310	78.2
**Fasting blood glucose** (mmol/L)	1676	4.81(1.55)

SD – standard deviation, SBP – systolic blood pressure, DBP – diastolic blood pressure, BMI – body mass index

### Prevalence of impaired fasting blood glucose and diabetes

The overall prevalence of diabetes was 4.8%. The prevalence rose with age up to 50–59 age group (12.5%, *P* < 0.001) (**Table 2**). The prevalence of diabetes was the highest in those widowed, divorced or separated (7.1%, *P* = 0.040). The prevalence decreased with education level, and was lowest among those with an education level of 3–year college and above (2.3%, *P* = 0.030). There was a significant difference in diabetes prevalence across participants with different occupation, being highest among those not working (7.7%, *P* = 0.021). Diabetes was 3 times more frequent in participants with hypertension than their counterparts (*P* < 0.001). There was a noteworthy 1.8–fold difference between the participants within different BMI groups (**Table 3**).

**Table 2:** Age–specific prevalence of impaired fasting blood glucose and diabetes

Age group	Impaired fasting blood glucose, No./n (%)	Diabetes, No./n (%)
18–	7/329 (2.1)	1/329 (0.3)
30–	20/617 (3.2)	10/617 (1.6)
40–	27/394 (6.9)	30/394 (7.6)
50–	30/232 (12.9)	29/232 (12.5)
60–70	17/103 (16.5)	11/103 (10.7)

**Table 3:** Prevalence of impaired fasting blood glucose and diabetes by socio–demographic and lifestyle characteristics

Characteristics	Impaired fasting blood glucose		Diabetes	
**No. (%)**	P*	**No. (%)**	P*
All participants	101 (6.0)	–	81 (4.8)	–
**Age group:**		<0.001		<0.001
18–44	43 (3.6)		28 (2.4)	
45–59	41 (10.5)		42 (10.7)	
≥60	17 (16.5)		11 (10.7)	
**Gender:**		0.087		0.860
Male	56 (7.1)		39 (4.9)	
Female	45 (5.1)		42 (4.7)	
Registration:		0.927		0.862
Locals	26 (5.9)		22 (5.0)	
Migrants	73 (6.0)		58 (4.8)	
Marital status:		0.245		0.040
Never in union	5 (3.4)		1 (0.7)	
Married or living with partner	90 (6.1)		76 (5.1)	
Widowed, divorced and separated	4 (9.5)		3 (7.1)	
**Education:**		0.011		0.030
Primary school and below	21 (9.1)		18 (7.8)	
Middle school	41 (6.8)		30 (5.0)	
High school and equivalent	29 (5.5)		24 (4.5)	
3–year college and above	8 (2.6)		7 (2.3)	
**Occupation:**		0.209		0.021
Manual workers	14 (5.5)		10 (4.0)	
Sales and services	22 (6.9)		13 (4.1)	
Professional, technical and managerial	9 (4.2)		9 (4.2)	
Clerical	7 (4.0)		3 (1.7)	
Other workers	11 (4.4)		10 (4.0)	
Not working	36 (7.9)		35 (7.7)	
**Household income:**		0.478		0.297
Low	11 (6.5)		10 (5.9)	
Middle	32 (5.3)		30 (5.0)	
High	17 (4.9)		11 (3.2)	
Rejected	14 (8.1)		6 (3.5)	
Do not know	27 (7.1)		24 (6.3)	
**Hypertension:**		<0.001		<0.001
Yes	32 (10.8)		32 (10.8)	
No	69 (5.0)		49 (3.5)	
**BMI:**		0.021		0.005
Underweight/normal weight	49 (4.9)		36 (3.6)	
Overweight/obese	52 (7.6)		45 (6.6)	
**Current smoking:**		0.219		0.849
Yes	27 (7.4)		17 (4.6)	
No	74 (5.6)		64 (4.9)	

BMI – body mass index

*χ^2^–test was used for comparisons.

The prevalence of impaired fasting blood glucose was 6.0%. The prevalence of impaired fasting blood glucose increased with age, and was highest among those aged ≥60 (16.5%, *P* < 0.001) (**Table 2**). Like the prevalence of diabetes, a similar trend was observed for impaired fasting blood glucose across participants with different education levels (*P* = 0.011). The prevalence of impaired fasting blood glucose among participants with hypertension was two times higher than that of their counterparts (10.8% vs 5.0%, *P* < 0.001). The prevalence of impaired fasting blood glucose in participants who were either overweight or obese was more prevalent when compared with their counterparts (7.6% vs 4.9%, *P* = 0.021) (**Table 3**).

The relationships between age and prevalence of diabetes and impaired fasting blood glucose were still statistically significant after adjusting for socio–demographic and lifestyle factors. However, the associations of other socio–demographic factors with prevalence of diabetes and impaired fasting blood glucose were non–significant after similar adjustments were made. The association with hypertension was significant for diabetes even after adjusting for socio–demographic and lifestyle factors (OR = 1.93, 95% CI 1.15, 3.22), whereas the association for impaired fasting blood glucose was not significant (OR = 1.48, 95% CI 0.91, 2.40). Significant relationships between BMI and diabetes (OR = 1.49, 95% CI 0.91, 2.43) and impaired fasting blood glucose (OR = 1.25, 95% CI 0.81, 1.93) were diminished after adjusting for confounding effects of socio–demographic and lifestyle factors (**Table 4**).

**Table 4:** Multivariate analysis on factors associated with prevalence of impaired fasting blood glucose and diabetes

Characteristics	Impaired fasting blood glucose		Diabetes	
OR (95% CI)*	**OR (95% CI)†**	OR (95% CI)*	**OR (95% CI)†**
**Age group:**				
18 – 44	1	1	1	1
45 – 59	2.76 (1.68–4.53)	2.51 (1.51–4.17)	3.48 (1.46–8.33)	2.78 (1.14–6.78)
≥60	4.72 (2.22–10.03)	4.17 (1.94–8.96)	4.15 (2.39–7.20)	3.54 (2.01–6.25)
**Gender:**				
Male	1.62 (1.02–2.57)	1.56 (0.93–2.65)	1.32 (0.78–2.24)	1.30 (0.72–2.37)
Female	1	1	1	1
**Registration:**				
Locals	0.98 (0.58–1.65)	0.92(0.52–1.65)	0.91(0.51,1.62)	0.92(0.52,1.65)
Migrants	1	1	1	1
**Marital status:**				
Never in union	1	1	1	1
Married or living with partner	2.07 (0.49–8.74)	1.97 (0.46–8.41)	3.21 (0.27–38.46)	2.97 (0.25–35.91)
Widowed, divorced or separated	1.22 (0.46–3.20)	1.16 (0.44–3.06)	3.98 (0.53–29.77)	3.62 (0.48–27.25)
**Education:**				
Primary school and below	2.11 (0.80–5.55)	2.01 (0.76–5.31)	1.23 (0.44–3.48)	1.13 (0.40–3.22)
Middle school	2.25 (0.95–5.36)	2.19 (0.92–5.20)	1.10 (0.42–2.84)	1.02 (0.40–2.64)
High school and equivalent	2.02 (0.87–4.70)	1.96 (0.85–4.57)	1.36 (0.54–3.41)	1.29 (0.52–3.25)
3–year college and above	1	1	1	1
**Occupation:**				
Manual workers	1.39 (0.60–3.18)	1.39 (0.61–3.21)	0.95 (0.37–2.46)	0.97 (0.38–2.51)
Sales and services	1.04 (0.42–2.54)	1.06 (0.43–2.62)	0.88 (0.32–2.40)	0.91 (0.33–2.49)
Professional, technical and managerial	1	1	1	1
Clerical	1.07 (0.38–3.02)	1.05 (0.37–2.97)	0.50 (0.13–1.99)	0.49 (0.12–1.95)
Other workers	0.70 (0.27–1.81)	0.71 (0.27–1.84)	0.85 (0.31–2.36)	0.88 (0.32–2.44)
Not working	1.05 (0.44, 2.49)	1.06 (0.45–2.54)	1.22 (0.48–3.09)	1.25 (0.49–3.19)
**Household income:**				
Low	0.89 (0.47–1.68)	0.88 (0.47–1.67)	1.46 (0.69–3.08)	1.44 (0.68–3.04)
Middle	1.05 (0.46–2.40)	1.07 (0.47–2.44)	1.45 (0.56–3.75)	1.51 (0.58–3.93)
High	1	1	1	1
Rejected	1.33 (0.59–3.00)	1.38 (0.61–3.13)	0.88 (0.28–2.70)	0.93 (0.30–2.88)
Do not know	1.01 (0.51–1.97)	0.98 (0.50–1.93)	1.25 (0.56–2.76)	1.20 (0.54–2.66)
**Hypertension:**				
Yes	–	1.48 (0.91–2.40)	–	1.93 (1.15–3.22)
No	–	1	–	1
**BMI:**				
Underweight/normal weight	–	1	–	1
Overweight/obese	–	1.25 (0.81–1.93)	–	1.49 (0.91–2.43)
**Current smoking:**				
Yes	–	0.99 (0.57–1.73)	–	0.89 (0.46–1.73)
No	–	1	–	1

OR – odds ratio, CI – confidence interval, BMI – body mass index

*Model adjusted for age, gender, marital status, registration, education, occupation and monthly household income.

**†**Model adjusted for age, gender, marital status, registration, education, occupation, monthly household income, hypertension, BMI and smoking status.

### Diabetes awareness, management and control

Among 81 participants with diabetes, 42 (51.9%) were aware of their condition. Among all participants with diabetes, 45.7% were treated with medications or insulin, while this percentage was 88.1% among participants with previously diagnosed diabetes. Dietary changes were adopted by 33.3% of participants, while 19.8% engaged in exercise and 23.5% monitored blood glucose regularly. Non-medical management approaches were almost two-fold more common in participants who were aware their condition: 64.3%, 38.1% and 45.2%, respectively. Only over one–tenth of the participants were under primary care management (**Table 5**).

**Table 5:** Awareness, management and control of diabetes

Variables	Among all patients with DM (No., %)	Among aware patients with DM (No., %)	Among patients with DM under drug treatment (No., %)
Awareness	51.9 (42/81)		
Management:			
Pharmacological	45.7 (37/81)	88.1 (37/42)	
Medications	39.5 (32/81)	76.2 (32/42)	
Insulin injection	6.2 (5/81)	11.9 (5/42)	
Non–pharmacological:			
Diet	33.3 (27/81)	64.3 (27/42)	
Exercise	19.8 (16/81)	38.1 (16/42)	
Blood glucose monitoring	23.5 (19/81)	45.2 (19/42)	
Under PC management	11.1 (9/81)	21.4 (9/42)	
Do not know	13.6 (11/81)	26.2 (11/42)	
Control	29.6 (24/81)	57.1 (24/42)	67.6 (25/37)

DM – diabetes mellitus, PC – primary care

## DISCUSSION

Our study on a representative sample of 1676 participants in Shenzhen, China, found that the prevalence of diabetes was 4.8%. The prevalence of impaired fasting blood glucose was 6.0%. The prevalence rates of both diabetes and impaired fasting blood glucose increased with age, whereas hypertension was strongly associated only with diabetes. The awareness of diabetes was poor and more than half of diabetic patients were not being treated pharmacologically. Less than one–third of diabetic patients were undertaking non–pharmacological treatments. Primary care management of diabetes was reported by only one–tenth of the participants.

This is a representative study with 1676 participants to investigate the epidemiology of diabetes in Shenzhen, China. A high response rate was achieved. Rigorous random sampling approach was adopted and implemented. Standard protocols and instruments were employed for blood pressure and blood glucose measurement. We followed the most commonly used international definition of the prevalence, awareness, treatment and control of diabetes to facilitate compatibility with the international literature. Data were collected by specially trained interviewers and supervised using a vigorous quality assurance program. However, the study had some limitations. First, the selection bias might have been introduced without knowing the characteristics of non–respondents, although the response rate was high. Second, data on awareness, pharmacological and non–pharmacological treatments, and primary care management were self-reported. We were not able to construct a criterion standard for rigid validation. Third, the diagnosis of diabetes was based on fasting blood glucose, which may underestimate the prevalence rates of diabetes and impaired fasting blood glucose. Fourth, we did not provide age and gender standardized estimate of prevalence of diabetes due to the unavailability of Shenzhen overall population information with respect to age and gender distribution. Therefore, caution is need for extrapolation of the findings. Last but not least, the cross–sectional nature of the current study does not allow establishing any causal relationships.

The overall diabetes prevalence in Shenzhen was 4.8%, which is in agreement with the 5.2% estimated by the Shenzhen Center for Chronic Diseases Prevention and Control [17]. However, our estimate is lower than that at the national level. The China National Diabetes and Metabolic Disorders Study showed that prevalence of diabetes was 9.7% between 2007 and 2008 [18]. The newest statics in 2010 indicated that the national average prevalence of diabetes was 11.6% [8]. Studies conducted in Beijing and Shanghai, which have economic context similar to that of Shenzhen, also yielded much higher prevalence rates, 9.0% [19] and 15.91% [20], respectively. Younger age of the participants in the current study, which was 39.26 on average, may help to explain the phenomenon, as studies have widely recognized the positive relationship between diabetes prevalence and age [21]. Un–implementation of oral glucose tolerance tests in the current study may have caused misclassifications and subsequent underestimation of diabetes prevalence in the current study [22]. However, it is impractical for large–scale epidemiological studies to adopt oral glucose tolerance tests for diagnosis of diabetes due to limited budget and time [23]. Although we observed lower prevalence of impaired fasting blood glucose than that at the national level (approximately 50% in 2010), its relatively higher prevalence rate than that of diabetes imposes a public health threat and burden to the health care system. Subjects with glucose impairment are at increased risk for developing diabetes, which indicates a substantially greater disease burden. Our findings highlight the importance of both primary and secondary prevention of diabetes, which challenges Shenzhen health care system’s capacity and capability.

Both the prevalence rates of impaired fasting blood glucose and diabetes increased with age, which is in line with the reported studies. A number of studies have shown that age is an important risk factor for diabetes [21]. However, the prevalence rate of diabetes in the current study had the peak in 50 to 59 age group, then a decreasing trend was observed, which is in conflict with the national study by Bragg et al. [1]. We also recorded higher prevalence of co–morbid hypertension among diabetic participants, which corresponds to previous reports [24]. Our finding implies that hypertension is a modifiable factor for diabetes, and public health efforts addressing diabetes should include shared, modifiable risk factors for several non–communicable diseases. Generally speaking, reducing blood pressure could reduce the risk of diabetes.

Overweight/obesity and smoking are well known to be closely associated with diabetes [25]. However, these associations were not observed in our study. Some participants may have changed their lifestyles after being diagnosed with diabetes, which might have influenced our results. Potential socioeconomic risk factors for diabetes, including poor education and low–income level, are not observed in the current study, and warrants further investigations.

We also showed that the management of diabetes was not optimism, especially the control rate. The diabetes awareness rate in our study was 51.9%, which is almost two times higher the national average in 2010 (30.1%) [8]. However, the awareness rate was lower than that in developed countries like the USA (72% in 2014) [26], which suggests a room for improvement. Although our study showed low pharmacological and non–pharmacological treatment rates in the general population of patients with diabetes, the pharmacological treatment rate was high (88.1%) among participants aware of their disease. Our findings comply with a previous study by Liu et al., which showed that drug treatment rate was 93.5% among diabetic patients who were aware their condition [21]. This implies that early screening may lead to the improvements in management and a decrease the subsequent complications and related social and disease burden. The establishment of health records for every community resident is a part of the national campaign and has been implemented across China, including Shenzhen. Documentation of blood glucose information for everyone may be an alternative for early detection of diabetes and pre–diabetes. Hypertension screening in primary care settings that has been performed in China, such as blood pressure tests for individuals aged ≥35–year who are at high risk of developing hypertension, may shed light on early detection of pre–diabetes or diabetes.

We found that primary care management of diabetes was just over one–tenth of all treatment modalities, although international and national studies have shown the relevance of primary care approach in managing chronic diseases [27,28]. Chronic diseases management is designed to be one of the six integrated services provided by primary care facilities. The Chinese government has also launched guidelines for standardized management of diabetes in primary care. Our study was not designed to test the relationship between primary care management and control rate of diabetes. Whether primary care standardized management of diabetes is an effective approach in reducing blood glucose needs further investigations.

## CONCLUSIONS

In conclusion, diabetes prevalence in Shenzhen (4.8%) is about half that of the Chinese average. Age and hypertension are the risk factors of diabetes in Shenzhen population. Approximately half of the subjects with diabetes are undiagnosed. Our findings highlight the need of public health efforts for primary and secondary prevention, as well as for early detection of diabetes. More attention should be paid to the residents aged between 50 and 59 years when formulating intervention strategies. Primary care may be relevant for an improved access to medical services and better management of diabetes.

## References

[1] Bragg F, Holmes MV, Iona A, Guo Y, Du H, Chen Y (2017). Association between diabetes and cause-specific mortality in rural and urban areas of China.. JAMA.

[2] World Health Organization. Global status report on noncommunicable diseases 2014. Geneva: WHO; 2014. 10.1161/STROKEAHA.115.008097 25873596

[3] WHO. Global health estimates: deaths by cause, age, sex and country, 2000-2012. Geneva: WHO; 2014.

[4] GBD 2013 Mortality and Causes of Death Collaborators (2015). Global, regional, and national age-sex specific all-cause and cause-specific mortality for 240 causes of death, 1990-2013: a systematic analysis for the Global Burden of Disease Study 2013.. Lancet.

[5] Zimmet PZ, Magliano DJ, Herman WH, Shaw JE (2014). Diabetes: a 21st century challenge.. Lancet Diabetes Endocrinol.

[6] National Diabetes Research Group (1981). Diabetes mellitus survey of 300,000 in fourteen provinces and cities of China.. Zhonghua Nei Ke Za Zhi.

[7] Liu S, Wang W, Zhang J, He Y, Yao C, Zeng Z (2011). Prevalence of diabetes and impaired fasting glucose in Chinese adults, China National Nutrition and Health Survey, 2002.. Prev Chronic Dis.

[8] Xu Y, Wang L, He J, Bi Y, Li M, Wang T (2013). Prevalence and control of diabetes in Chinese adults.. JAMA.

[9] Shen H. Effectiveness of a peer-led self-management program for older people with type 2 diabetes in China [dissertation].Brisbane: Queensland University of Technology; 2008.

[10] Williams ED, Magliano DJ, Zimmet PZ, Kavanagh AM, Stevenson CE, Oldenburg BF (2012). Area-level socioeconomic status and incidence of abnormal glucose metabolism: the Australian Diabetes, Obesity and Lifestyle (AusDiab) study.. Diabetes Care.

[11] Yach D, Stuckler D, Brownell KD (2006). Epidemiologic and economic consequences of the global epidemics of obesity and diabetes.. Nat Med.

[12] Agardh E, Allebeck P, Hallqvist J, Moradi T, Sidorchuk A (2011). Type 2 diabetes incidence and socio-economic position: a systematic review and meta-analysis.. Int J Epidemiol.

[13] Riley L, Guthold R, Cowan M, Savin S, Bhatti L, Armstrong T (2016). The World Health Organization STEPwise approach to noncommunicable disease Risk-Factor Surveillance: methods, challenges, and opportunities.. Am J Public Health.

[14] Li H, Chung RY, Wei X, Mou J, Wong SY, Wong MC (2014). Comparison of perceived quality amongst migrant and local patients using primary health care delivered by community health centres in Shenzhen, China.. BMC Fam Pract.

[15] Shenzhen Statistics Bureau, NBS Survey Office in Shenzhen. Shenzhen Statistical Yearbook 2015. Beijing: China Statistics Press; 2015.

[16] Yang W, Li H, Fu X, Lu J, Xue Z, Wu C (2015). Inequalities in cardiovascular health between local and migrant residents: a cross-sectional study of 6934 participants in Longhua District, Shenzhen.. Medicine.

[17] Zhou HB, Peng J, Liu XL, Lin HC, Zhang D (2011). Prevalence of diabetes in Shenzhen between 1997 and 2009.. Zhonghua Yu Fang Yi Xue Za Zhi..

[18] Yang SH, Dou KF, Song WJ (2010). Prevalence of diabetes among men and women in China.. N Engl J Med.

[19] People’s Government of Beijing Municipality. Health and population health status report of Beijing city in 2011. [book in Chinese]. Beijing: People's Medical Publishing House; 2012.

[20] Qin Y, Wang R, Ma X, Zhao Y, Lu J, Wu C (2016). Prevalence, awareness, treatment and control of diabetes mellitus-a population based study in Shanghai, China.. Int J Environ Res Public Health.

[21] Liu X, Li Y, Li L, Zhang L, Ren Y, Zhou H (2016). Prevalence, awareness, treatment, control of type 2 diabetes mellitus and risk factors in Chinese rural population: the RuralDiab study.. Sci Rep..

[22] Silva-Matos C, Gomes A, Azevedo A, Damasceno A, Prista A, Lunet N (2011). Diabetes in Mozambique: prevalence, management and healthcare challenges.. Diabetes Metab.

[23] Motala AA, Omar MA, Pirie FJ (2003). Diabetes in Africa. Epidemiology of type 1 and type 2 diabetes in Africa.. J Cardiovasc Risk..

[24] Stanifer JW, Cleland CR, Makuka GJ, Egger JR, Maro V, Maro H (2016). Prevalence, risk factors, and complications of diabetes in the Kilimanjaro Region: a population-based study from Tanzania.. PLoS One.

[25] World Health Organization. Obesity and overweight. 2011. Available: http://www.who.int/mediacentre/factsheets/fs311/en/. Accessed: 5 June 2016.

[26] Centers for Disease Control and Prevention. 2014 National Diabetes Statistics Report. 2014. Available: https://www.cdc.gov/diabetes/pubs/statsreport14/national-diabetes-report-web.pdf. Accessed: 8 June 2016.

[27] Li H, Wei X, Wong MC, Yang N, Wong SY, Lao X (2015). A comparison of the quality of hypertension management in primary care between Shanghai and Shenzhen: a cohort study of 3196 patients.. Medicine.

[28] Hammouche S, Holland R, Steel N (2011). Does quality of care for hypertension in primary care vary with postcode area deprivation? An observational study.. BMC Health Serv Res.

